# Recurrent visceral leishmaniasis in an immunocompetent patient: a case report

**DOI:** 10.1186/1752-1947-7-68

**Published:** 2013-03-14

**Authors:** Maria Lagadinou, Dimitra Dimitropoulou, Stelios F Assimakopoulos, George Davoulos, Markos Marangos

**Affiliations:** 1 Department of Internal Medicine, University Hospital of Patras, Rion-Patras 26504, Greece; 2 Division of Infectious Diseases, Department of Internal Medicine, University Hospital of Patras, Rion-Patras 26504, Greece

**Keywords:** Leishmaniasis, Recurrent, Immunocompetent, Liposomal amphotericin B

## Abstract

**Introduction:**

Current treatment options for visceral leishmaniasis (pentavalent antimony, amphotericin B, liposomal amphotericin B and mitelfosine) achieve long-term clinical cure in the majority of immunocompetent patients. Disease relapse is usually provoked by T-cell number or function impairment (corticosteroid or cytotoxic therapy, transplant recipients, advanced human immunodeficiency virus disease).

**Case presentation:**

We report a case of visceral leishmaniasis with multiple relapses in a 75-year-old Greek immunocompetent man. Visceral leishmaniasis relapses occurred despite appropriate treatment with liposomal amphotericin B 3mg/kg/day on days one to five, 14 and 21 (for the first episode and the first relapse) and mitelfosine 150mg/day for 28 days (for the second relapse). The third relapse was treated with high-dose liposomal amphotericin B (10mg/kg for two consecutive days), followed by a secondary prophylaxis of 3mg/kg once per month, which prevented disease reappearance during one year of follow-up.

**Conclusion:**

An unusual case of recurrent visceral leishmaniasis in an older immunocompetent patient was treated with high-dose liposomal amphotericin B and a monthly prophylaxis with no evidence of a relapse after one year of follow-up.

## Introduction

Leishmaniasis is a globally widespread group of parasitic diseases caused by different species of parasites that belong to the genus *Leishmania*. It is a zoonosis and the parasite is transmitted by the bite of an infected female phlebotomine sand fly. Clinically, it is subdivided into visceral (kala-azar), cutaneous and mucocutaneous forms. Visceral leishmaniasis (VL), the most severe form, is a disseminated intracellular protozoan infection that targets tissue macrophages in the liver, spleen and bone marrow. *Leishmania donovani* and *Leishmania infantum* can both cause VL, but *L. infantum* is the predominant pathogen in Mediterranean countries [1,2].

The outcome and clinical features of the disease depend on both parasite properties (infectivity, pathogenicity, virulence) and host factors (age, nutritional status, and efficacy of innate and cell-mediated immunity). With appropriate treatment, long-term clinical cure is usually achieved; however, eradication of parasites is unlikely in most patients, as evidenced by the fact that, despite initial clinical cure, the disease can reactivate several years later and parasites can be persistently detected with application of sensitive assay methods like polymerase chain reaction [3]. Disease relapse occurs frequently in patients with T-cell number or function impairment (corticosteroid or cytotoxic therapy, transplant recipients, advanced human immunodeficiency virus disease). Rarely, spontaneous reactivation can occur in immunocompetent patients, attributed mainly to drug resistance or suboptimal treatment regimens [4,5].

Here we report the case of an older immunocompetent patient who had multiple VL relapses, despite appropriate treatment with liposomal amphotericin B (L-AmB) and mitelfosine. His relapsing disease course was attributed to advanced age-induced immunosuppression as no other cause was identified.

## Case presentation

A 75-year-old Greek man, resident in an urban area, presented to our hospital complaining of high fever with chills and rigors, night sweats, fatigue and weight loss of 3kg in one month. His previous medical history was unremarkable. A physical examination revealed a moderately enlarged spleen.

Laboratory investigation showed leukopenia (leukocytes: 2100/μL), normochromic-normocytic anemia (hemoglobin: 11.3g/dL) and moderate thrombocytopenia (platelets: 101,000/μL). A high erythrocyte sedimentation rate (ESR) (90mm/hour) and C-reactive protein (4mg/dL) were also noticed. The biochemical tests were normal except for elevated serum globulins (4.2g/dL). The serum protein electrophoresis revealed a polyclonal γ-globulin pattern without detection of a monoclonal component. His chest X-ray and urine analysis were normal. His blood and urine culture results were repeatedly negative. A transthoracic echocardiogram revealed no pathology and his tuberculin skin test result was negative. An abdominal computed tomography scan with contrast medium revealed the presence of a few hyperechogenic splenic lesions. Serology results for hepatitis A, B, and C, Coxsackie virus, enteric cytopathic human orphan virus, herpes simplex virus, Epstein-Barr virus, cytomegalovirus, human immunodeficiency virus, human T-lymphotropic virus type 1 and 2, toxoplasma, *Bartonella henselae*, *Francisella tularensis*, *Coxiella burnetii* and *Rickettsia conorii* were negative. Wright and rapid plasma reagin tests were also negative. A full immunological screening with rheumatoid factor, antinuclear antibodies, antibodies to double-stranded deoxyribonucleic acid (DNA), anti-Sm, anti-Ro/SSA, anti-La/SSB, anti-ribonucleoproteins, anti-Jo-1, anti-Scl-70, anti-histones, anti-mitochondrial antibodies, anti-smooth muscle antibodies, centrally accentuated antineutrophil cytoplasmic antibodies (c-ANCA), perinuclear antineutrophil cytoplasmic antibodies (p-ANCA), anti-cardiolipin, and lupus anticoagulant was negative. A bone marrow aspiration showed numerous protozoan parasites (Leishmanian spp.) within macrophages. Serum test results for leishmania antibodies and K39 antigen were positive.

After confirming the diagnosis of VL, the patient was treated with L-AmB 3mg/kg/day on days one to five, 14 and 21 intravenously. His tolerance of amphotericin was excellent with no significant changes in his kidney and liver functions. Treatment was completed uneventfully and was associated with prompt remission of the patient’s symptoms in conjunction with a gradual recovery of leukocyte and platelet counts and hematocrit level.

Six months later, he reported intermittent fever and deterioration of his health. The laboratory tests showed leukopenia, anemia and thrombocytopenia again. The physical examination was normal. His blood and urine cultures were still negative and a new abdominal computed tomographic scan showed no pathological findings. A bone marrow smear revealed the presence of leishmania amastigotes, though in much lower numbers than those detected six months previously. The patient received another complete course of intravenous L-AmB at the same dose, which resulted in complete clinical and laboratory remission.

However, three months later, the patient had to be hospitalized again for a new episode for VL that was confirmed with a new bone marrow smear. The laboratory and serological findings were compatible with the previous results. A total lymphocyte count and CD4+ percentage, as well as their absolute count, were measured in order to evaluate the immune status of our patient. CD4+ cell percentage and absolute count were found to be relatively low (22% and 450/μm^3^, respectively). Mitelfosine (Impavido® 50mg/Zentaris GmbH, Germany) was administrated in a dosage of 150mg per day (three capsules once daily) for 28 days. Treatment was generally well tolerated; gastrointestinal symptoms such as vomiting and anorexia were controlled with a split of the daily dose into two doses, which were co-administered with food.

Three months later, the patient required another hospitalization for irregular fever, sweats and fatigue. The laboratory tests once again revealed pancytopenia. His test results for leishmania antibodies and K39 antigen were positive. The patient was reluctant to be subjected to an additional bone marrow biopsy. However, there was a strong clinical evidence for a new episode of VL, therefore a new course of L-AmB administration was decided in a dose of 10mg/kg on days one to two, in combination with oral mitelfosine. Unfortunately, the tolerance of mitelfosine was very poor this time because of vomiting; this led the patient to stop the drug three days after initiation. Nevertheless, the administration of high-dose L-Amb resulted in complete clinicolaboratory response. In order to prevent further disease relapses, a secondary prophylaxis with L-AmB once per month at a dose of 3mg/kg was scheduled. Currently, almost a year after the last VL episode, the patient remains in good health with normal hematological indices.

## Discussion

Leishmania organisms are flagellated protozoa that are usually transmitted by female phlebotomine sand flies to mammalian hosts, where they reproduce as obligate intracellular parasites in the mononuclear phagocytic system. The cardinal pathogenetic characteristics of leishmania infection are: (i) the parasite replicates intracellularly in resident tissue macrophages, (ii) the host immune-inflammatory response regulates the expression and outcome of the disease and (iii) tissue infection is usually persistent [6]. Dissemination depends on different factors such as parasite properties, host factors and host responses. Ideally, after promastigotes enter macrophages, the immunocompetent host develops nonspecific (innate) and cell-mediated immunity in order to eradicate the infection, leading to spontaneous healing and prevention of reactivation. Secretion of proinflammatory Th1-type cytokines such as interferon-γ, interleukin-12 and tumor necrosis factor-α prevents parasitemia and development of chronic disease. On the contrary, in immunosuppressed patients, immune responses are ineffective and dissemination of disease occurs frequently [7].

Current therapies for VL, such as pentavalent antimony, amphotericin B, L-AmB and mitelfosine, lead to clinical cure in the majority of immunocompetent patients. Recurrent infections occur within a period of six months, commonly in patients with impaired cellular immunity such as individuals with advanced human immunodeficiency virus disease or immunosuppressed transplant recipients [8].

In this article, we report on multiple episodes of relapsing VL in an older patient. Beyond the known decreased capacity of cytotoxic responses during senescence, no other immune-suppressing factor was identified in our case. CD4+ cell percentage and absolute count measured in the second VL relapse were found to be relatively low (22% and 450/μm^3^, respectively). However, even in human immunodeficiency virus cases with CD4+ cell counts reaching 250 to 300/μm^3^ the risk of relapse seems to be low [9]. To the best of our knowledge, there are very few reports of relapsing VL in the immunocompetent in the literature [10].

Initially, our patient was treated with L-Amb and the same regimen was given in his first episode of relapse. L-AmB is considered a highly effective regimen for VL treatment even in immunosuppressed patients [11,12]. However, this treatment option failed to succeed in chronic suppression of the disease. Mitelfosine, an alkylphosphocholine with antileishmanial activity, was used as an alternative treatment in his second episode of relapse. Studies in immunocompromised patients (human immunodeficiency virus seropositive) have shown that the antileishmanial activity of this drug is retained in this special population, suggesting that mitelfosine might be a useful option in preventing relapses of the disease [13]. Unfortunately, even after mitelfosine therapy, our patient had a new episode of VL.

L-AmB shows a concentration-dependent activity against Leishmania spp, where higher doses of L-AmB result in a greater reduction of the parasite load and in a persistence of drug levels in the liver and spleen for a long period after cessation of therapy [11]. In order to eradicate the infection, we decided to administer the high dose and shorter duration scheme of L-AmB (10mg/kg for two consecutive days) with a subsequent secondary prophylaxis. Current evidence in acquired immune deficiency syndrome-related VL suggests that maintenance treatment with L-AmB at a dose of 3mg/kg once every three or four weeks may suppress recurrent infection, probably by inhibition of the reproduction of the remaining parasites, which represent an endogenous reservoir responsible for future relapses [14,15]. Our patient showed an excellent tolerance to therapy with no renal or electrolyte disturbances and exhibited a complete clinical and laboratory response.

## Conclusions

An unusual case of recurrent VL in an older immunocompetent patient was effectively treated with high-dose L-AmB and a monthly prophylaxis with no evidence of relapse after one year of follow-up. High-dose and shorter duration regimens of L-AmB seem to be a well-tolerated therapy in older patients, while maintenance therapy may be needed in some cases in order to prevent future VL relapses.

## Consent

Written informed consent was obtained from the patient for publication of this case report and accompanying images. A copy of the written consent is available for review by the Editor-in-Chief of this journal.

## Abbreviations

ANCA: antineutrophil cytoplasmic antibody; ESR: erythrocyte sedimentation rate; HIV: human immunodeficiency virus; L-AmB: liposomal amphotericin B; VL: visceral leishmaniasis.

## Competing interests

The authors declare that they have no competing interests.

## Authors’ contributions

ML and DD wrote this case report. ML, DD, SFA, GD and MM were the patient’s doctors. SFA and MM critically revised the manuscript. All authors have read and approved the final version of this manuscript.
